# GPR17 in Glioblastoma: Structure, Ligand Interactions, and Therapeutic Targeting

**DOI:** 10.1021/acsomega.5c04079

**Published:** 2025-09-29

**Authors:** Ramalakshmi Satyanarayana, Sree Somala Chaitanya, Iswarya Suresh Kumar, Ramesh Thiyagarajan, Sureka Chandrabose, Kasim S. Abass, Saravanan Konda Mani, Anand Thirunavukarasou, Meenakshisundaram Kandhavelu

**Affiliations:** † Faculty of Clinical Research, 204733 Sri Ramachandra Institute of Higher Education and Research, Chennai, Tamil Nadu 600116, India; ‡ B Aatral Biosciences Private Limited, Bengaluru, Karnataka 560091, India; § Department of Basic Medical Sciences, College of Medicine, 204568 Prince Sattam Bin Abdulaziz University, Al-Kharj 11942, Saudi Arabia; ∥ Department of Physiology, Biochemistry, and Pharmacology, College of Veterinary Medicine, University of Kirkuk, Kirkuk 36003, Iraq; ⊥ Molecular Signaling Group, Faculty of Medicine and Health Technology, 7839 Tampere University, Tampere 33101, Finland; # BioMeditech and Tays Cancer Center, Tampere University Hospital, Tampere 33014, Finland

## Abstract

G protein-coupled receptor 17 (GPR17) is a crucial protein encoded by the GPR17 gene, which belongs to the G protein-coupled receptor (GPCR) family. It serves a pivotal function in cellular responses to various stimuli. GPR17 is found in various organs, including the brain, spinal cord, kidneys, liver, and immune cells. It is especially prevalent in oligodendrocytes, underscoring its significance in myelination. GPR17 is involved in myelination, inflammation, and neuroprotection. Recent studies highlight the therapeutic potential of targeting GPR17 in glioblastoma, a highly aggressive brain cancer, as it is overexpressed in tumor tissues and plays a critical role in tumor progression and invasion. Understanding the structure of GPR17 and its interactions with ligands and functional signaling pathways is crucial for developing targeted therapeutics for conditions involving myelin degradation, neuroinflammation, and immune dysregulation. To guide this exploration, this review is organized into distinct sections covering the sequence and structural features of GPR17, its natural and synthetic ligands, its role in glioblastoma progression, its associated signaling pathways, and the potential of using GPR17 as a therapeutic target. Each section in the review consolidates current findings to offer an integrated view of GPR17 biology and its translational relevance in oncology.

## Graphical abstract

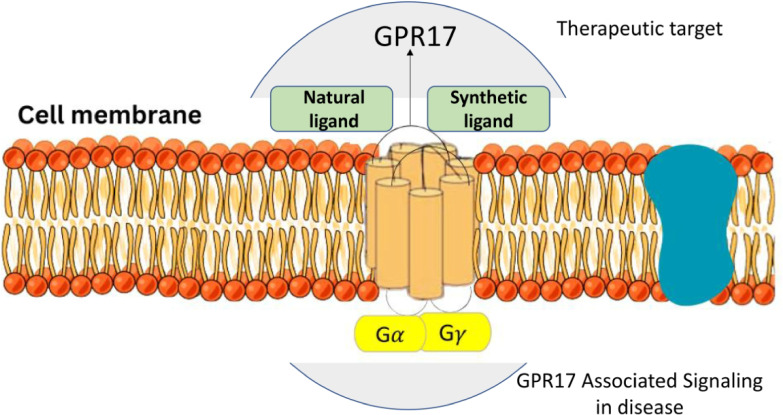

## 1 Introduction

GPR17 is an orphan receptor within the G protein-coupled receptor (GPCR) family, which is crucial in transducing extracellular signals into intracellular responses.^1^ These receptors play a vital role in various physiological domains, including sensory perception, hormonal activity, and neuronal transmission.^2^ GPR17, cloned in the late 1990s, gained interest due to its role in regulating metabolism and neurobiology, thereby influencing processes including oligodendrocyte development.^3^ GPR17 was initially recognized as an orphan receptor and further studies validated its affinity for uracil nucleotides and cysteinyl leukotrienes, yet the evidence remains inconclusive and is the subject of current discourse.^1,4^

Reports demonstrate the presence of GPR17 protein anatomically, including the brain, spinal cord, kidneys, liver, and immune cells, suggesting its role in multiple physiological processes.^5^ Oligodendrocytes in the central nervous system are responsible for the synthesis of the myelin sheath, which insulates axons and plays a crucial role in nerve conduction and repair.^6^ GPR17 functions as a negative regulator of oligodendrocyte differentiation, maintaining oligodendrocyte precursor cells (OPCs) in an undifferentiated state and thereby inhibiting premature myelin formation.^7^ Thus, disruption in GPR17 signaling is associated with various demyelinating and inflammatory conditions in the nervous system, including multiple sclerosis.^8,9^

Clinical research has shown that certain FDA-approved drugs, like cangrelor and montelukast, typically inhibit the functions of GPR17. Considering cangrelor’s mechanism of action, it is possible that inhibiting GPR17-associated pathways could regulate microglial activation and the related neuroinflammation involved in Glioblastoma multiforme (GBM).^10^ Similarly, montelukast, a prevalent asthma drug that antagonizes both its primary target and GPR17, helps in reducing the leukotriene-mediated inflammatory response associated with asthma.^11^ By inhibiting GPR17, these drugs may reduce tumor-associated inflammation and impede glioma progression. The diverse applications of these drugs suggest that targeting GPR17 may provide significant advantages for the treatment of GBM and necessitate further investigation. GPR17 has been recognized as a potential therapeutic target due to the discovery of its ligands and its associated pathways.^12^ This review demonstrates the role of GPR17 research in GBM, an aggressive and lethal brain cancer characterized by rapid proliferation, unfavorable prognoses, and treatment challenges.^13^ It was identified that GPR17 is significantly overexpressed in GBM when compared to normal brain tissue.^14^ Elevated GPR17 levels in GBM lead to increased tumor cell invasion, proliferation, treatment resistance, and worse prognostic indicators for the patient.^15,16^

Research involving specific methods indicates that the activation of GPR17 by UDP and CysLTs promotes GBM progression through enhanced cellular motility, increased proliferation, and the epithelial-mesenchymal transition (EMT), hence advancing tumor development and complicating treatment strategies.^17^ This suggests that GPR17 enables GBM cancer cells to evade therapeutic interventions and infiltrate other brain regions. Based on these findings, identifying GPR17 as a therapeutic target for GBM is a viable alternative. Several in vitro and in vivo studies by our team have shown that GPR17 agonists can modulate receptor activity, resulting in decreased cell proliferation, migration, and invasion of GBM,^16,19,20^ It is crucial to contextualize the genetic modifications and molecular pathways influencing glioblastoma, considering the importance of GPR17 in GBM progression and its potential as a therapeutic target.

## 2 Therapeutic Targets for GBM

A comprehensive genome-wide study of more than 20,000 genes from 22 glioblastoma tumor genomes found the majority of mutations that are likely to promote glioblastoma development.^20^ These DNA modifications include point mutations, substantial copy-number changes, minor insertions and deletions (genomic amplifications and deletions), leading to tumor formation most frequently.^21^ Examination of the altered genes revealed that they regulate the significant pathways involved in cell development, cell cycle regulation, and other processes. DNA mutation leads to cancer, causing alterations in gene transcription patterns.^22^

Although GBM often does not spread to other organs, it aggressively invades brain tissue.^23^ Growth and invasion need manipulation of the microenvironment, and various aspects of this may be the focus of GBM treatments. A crucial factor in the GBM microenvironment is the transforming growth factor (TGF), which supports tumor cells to behave more aggressively and promotes their survival, preventing host suppression.^24^ The most numerous and varied class of integral membrane proteins in eukaryotes serves as an attractive pharmacological target for different disorders that mediate various signaling events. Despite having seven transmembrane helices, GPCRs possess highly variable loops that perform a range of physiological tasks. When small-molecule ligands activate the transmembrane helices in the receptor’s extracellular domain, the carboxyl-terminal of the receptor, in conjunction with the guanine (G) protein, triggers the critical signaling pathways.^25,26^ Humans have been shown to have around 1347 GPCR structures, which have been categorized into six main classes and 69 subfamilies (including receptor complexes) and stored in GPCRdb.^27^ The GPCR-EXP public repository, which contains experimentally solved and predicted structures, facilitated the therapeutic assessment through computational approaches.^28^ Several studies have indicated a strong connection between glioma-initiating cells and OPCs, suggesting that specific variants of GBM may originate from or exhibit characteristics of OPCs.^29^ GPR17 is mainly located in OPCs and plays a crucial role in regulating their development and response to damage. Consequently, GPR17 represents a promising target for GBM treatment, as it is implicated in brain disorders and contributes to GBM development and tumor proliferation.

GPR17 is associated with two distinct receptor families, P2Y and CysLT, for UDP and CysLTs, respectively, both physically and phylogenetically related.^30^ Over 60% of currently marketed pharmaceuticals target P2Y receptors, a subset of seven-transmembrane (TM) rhodopsin family G protein-coupled receptors (GPCRs). Our group comparatively elucidated the functional role of GPR17 targeting P2Y1 receptor, a subclass of P2Y receptors.^18^ The atomic structure of GPR17 has facilitated the computational elucidation of the ligand-binding site, essential for comprehending its molecular mechanism of action.^31^ Understanding the structural and functional characteristics of GPR17 not only highlights its therapeutic relevance but also paves the way for exploring its role in tumorigenesis, especially in glioblastoma.

## 3 Role of GPR17 in Glioblastoma and the Central Nervous System

The investigation of GPR17 uncovers a protein structure consisting of seven transmembrane domains linked by extracellular and intracellular loops. The primary structure of GPR17 exhibits a high level of conservation across different species, indicating its evolutionary and functional significance.^32^ The potential phosphorylation sites regulate the receptor activity and initiate downstream signaling cascades.^33^ GPR17 and its sequence features comprise many structural and functional components necessary for its function.^34^ One crucial feature of GPR17’s sequence is that it has transmembrane domains. GPR17 has seven transmembrane regions (TM1–TM7) that traverse the lipid bilayer of the cell membrane. These transmembrane regions are crucial for maintaining the receptor’s position in the membrane and facilitate ligand binding and signal transmission.^35^ Extracellular loops (ECLs) and intracellular loops (ICLs) connect the transmembrane regions. The GPR17 extracellular loops contain regions that interact with ligands, including neurotransmitters, hormones, or other signaling molecules. On the other hand, the intracellular loops connect GPR17 to intracellular signaling pathways by interacting with G proteins and other signaling molecules. These loops also help reduce, internalize, and recycle receptors.^36^

The GPR17 sequence contains specific amino acid residues that are conserved across various species, indicating a significant role in its function. These conserved residues are often located within the transmembrane domains and contribute to ligand binding, receptor activation, and downstream signaling events.^37^ GPR17 exhibits distinctive sequence patterns characteristic of G protein-coupled receptors, including the Asp-Arg-Tyr motif located in the second extracellular loop.^38,39^ This motif plays a crucial role in the coupling and activation of G proteins. Sequence study of GPR17 identifies possible phosphorylation sites for protein kinases, including serine and threonine residues found in the internal loops and C-terminal end. The phosphorylation of these sites regulates receptor activation, desensitization, and internalization. Analyzing the sequence features of GPR17 provides valuable insights into its structural arrangement, ligand interactions, and signaling pathways.

Until October 2022, the three-dimensional (3D) structure of GPR17 had not been determined using experimental techniques such as nuclear magnetic resonance (NMR) spectroscopy and X-ray crystallography.^31^ Computational modeling approaches, including homology modeling and molecular dynamics simulations, have been employed to develop theoretical models of GPR17.^13,16,18^ These models are based on the structures of closely related GPCRs with known crystal structures. These models offer crucial insights into the probable structure and functional mechanisms of GPR17. Although the structure of GPR17 has not been determined experimentally, computational models have identified possible binding sites for natural ligands, such as nucleotides and leukotrienes, within the transmembrane bundle and extracellular loops. These models also suggest structural changes occur when the receptor is activated and connected to intracellular signaling pathways through heterotrimeric G proteins.^42^

Ye et al. employed cryo-electron microscopy to elucidate the structure of the activated GPR17-Gi complex^31^ at a resolution of 3.02 Å, and further mutagenesis experiments suggested that extracellular loop 2 of GPR17 occupies the orthosteric binding pocket, promoting its self-activation. The GPR17 receptor in its active state had several microswitches typically observed in other class A GPCRs. Additionally, the Gi protein interacts with the essential amino acid residues of transmembrane helix 3 (TM3), the amphipathic helix 8 (Helix 8), and intracellular loop 3 (ICL3) in GPR17, establishing a connection within the receptor core.^31^ The structure thus highlights the activation mechanism of GPR17 based on its structural characteristics, which will ultimately help in the development of pharmacological therapies for acute or chronic central nervous system injuries.

In the context of the purinergic receptor group, GPR17 is further elucidated by its tight association with P2Y2R, P2Y1R, and P2Y12R, all of which respond to extracellular stimuli.^39^ All four are members of the GPCR family and possess a shared structure characterized by seven transmembrane segments. In contrast to P2Y1R and P2Y12R, which respond only to adenine nucleotides, GPR17 is activated by uracil nucleotides and cysteinyl leukotrienes. While P2Y2R recognizes both ATP and UTP, it does not respond to leukotrienes, thereby designating GPR17 as a dual-sensitive receptor.^18^ P2Y receptors are primarily significant for platelet aggregation (P2Y12R), regulating vascular tone (P2Y1R), and facilitating ion transport in the epithelium (P2Y2R). Conversely, the majority of GPR17’s actions in the body pertain to its role in the central nervous system, encompassing oligodendrocyte differentiation, enhancement of myelin formation, and regulation of neuroinflammation.^43^ GPR17’s distinctive characteristic features, particularly in its interactions with external stimuli, render it an exceptional target for novel therapeutics in the treatment of cancer. GPR17, being a purinergic receptor with its unique ligand specificity, is crucial to investigate how its interactions with both natural and synthetic ligands affect receptor activation and downstream signaling pathways.

## 4 Pharmacology of GPR17

### 4.1 Natural Ligands

GPR17 has been observed to bind to many ligands. Table 1 presents a curated list of natural and synthetic ligands known to interact with GPR17, categorized by their origin and type.^5,44^ These interactions are crucial in regulating GPR17 activation and the subsequent signaling cascades.^45^ While multiple listed compounds are considered natural ligands of GPR17, it is important to note that some, such as UDP and cysteinyl leukotrienes (LTC_4_, LTD_4_, LTE_4_), have been more extensively studied in the context of receptor activation and functional relevance. Researchers emphasize uridine diphosphate (UDP) and UDP-glucose, as they function partially as agonists to regulate receptor activation. ATP is well-known for its interaction with P2X and P2Y receptors; however, GPR17 has been shown to respond to nucleotide derivatives such as UDP and UDP-glucose, indicating a broader ligand recognition profile among purinergic receptors.^46^ Molecules known as LTE4, LTC4, and LTD4, derived from lipids, activate GPR17 and facilitate inflammatory and immunological responses.^47^ The ligands exhibit variations in binding sites and downstream signaling events, where nucleotide agonists primarily induce intracellular calcium release,^11^ while leukotrienes facilitate receptor removal and deactivation. Due to its dual-ligand specificity, GPR17 may be implicated in several conditions, including cerebral inflammation and brain malignancies. UDP-glucose, a compound derived from UDP, functions as an agonist for GPR17, but its efficacy is lower than that of UDP.^48^

**Table 1:** List of Natural and Synthetic Ligands Reported to Bind GPR17^a^

S. No	Ligand	Type	Compound ID	PubMed Reference
1	ATP	Natural	1713	21773766
2	LTE4, LTD4, LTC4	Natural	3352, 3353, 3354	18974869
3	UDP	Natural	1749	16990797, 20148890
4	UDP-galactose	Natural	1782	18974869
5	UDP-glucose	Natural	1783	18974869
6	SDF-1 (CXCL12)	Natural	1QG7	24613411
7	27-hydroxycholesterol	Natural	123976	36214386
8	Cangrelor	Synthetic/Repurposed antagonist	9854012	19625605
9	Montelukast	Synthetic/Repurposed antagonist	5281040	16990797
10	MRS 2179	Synthetic antagonist	5311302	18533035
11	N6-cyclopentyl ATP	Synthetic antagonist	657378	21744154
12	ASN02563583	Synthetic agonist	5517	21744154
13	ASN04885796	Synthetic agonist	5520	21744154
14	ASN06917370	Synthetic agonist	5524	21744154
15	MDL 29951	Synthetic agonist	446916	27733608
16	Galinex	Synthetic agonist	10817	32320409
17	CHBC (Indoline derivative)*	Synthetic agonist	CHBC	35016881
18	AC1MLNKK*	Synthetic agonist	AC1MLNKK	28827203
19	T0510.3657*	Synthetic agonist	3516396	28827203
20	24(S)-hydroxycholesterol (24S-HC)	Synthetic agonist	SML1648	36214386
21	HAMI3379	Synthetic/Repurposed antagonist	10438479	29706593

a Ligands marked with * were identified and/or synthesized by the authors’ research group. A reference column is included to help readers locate the primary studies supporting the activity of each compound at GPR17.

Figure 1 illustrates the diversity of endogenous ligands that bind to GPR17, categorizing them into conventional and nonconventional types. GPR17 is also activated by previously mentioned cysteinyl leukotrienes, specifically leukotrienes C4 (LTC4) and D4 (LTD4), which contribute to its dual ligand recognition profile. These ligands are lipid mediators that originate from the metabolism of arachidonic acid and play a role in inflammatory and immunological responses.^49^ GPR17 is activated by LTC4 and LTD4, releasing intracellular calcium and suppressing cAMP synthesis, a process comparable to that induced by UDP binding.^50^ GPR17 may interact with additional endogenous or exogenous ligands other than UDP, UDP-glucose, LTC4, and LTD4.^51^ Further investigation is required to identify and describe more ligands for GPR17 and clarify their functions in activating the receptor and initiating subsequent signaling processes. Understanding the interactions between GPR17 and its ligands is crucial for elucidating the receptor’s physiological functions and developing therapeutic strategies targeting GPR17 in various diseases, including neurological disorders and inflammatory conditions.

**Figure 1:**
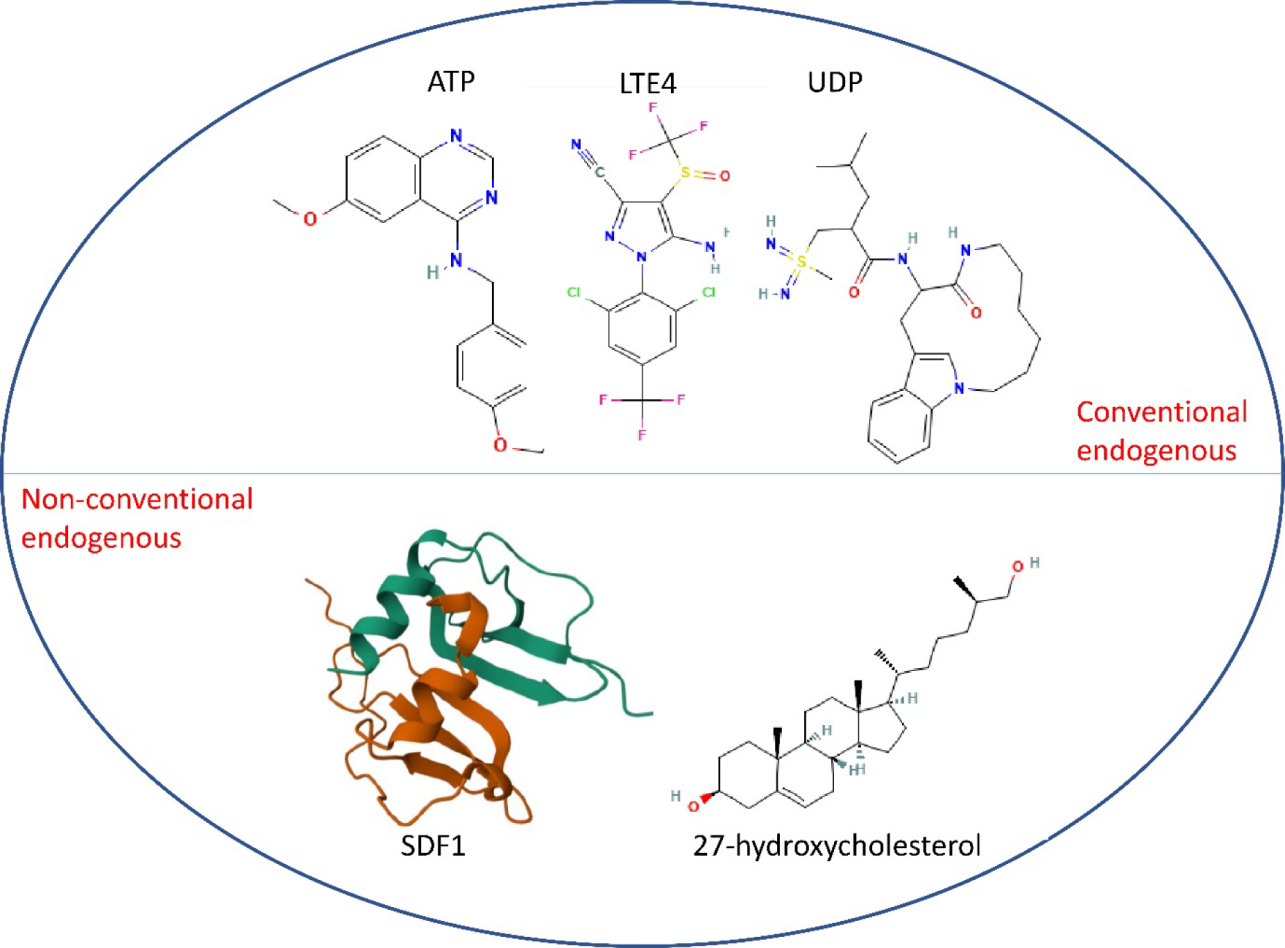
Representative structures of GPR17 endogenous ligands. The upper panel shows conventional endogenous ligands (ATP, LTE4, and UDP) commonly associated with classical GPCR signaling. The lower panel displays nonconventional endogenous ligands (SDF1 (a chemokine) and 27-hydroxycholesterol), highlighting alternative pathways through which GPR17 may be activated in physiological and pathological contexts.

### 4.2 Synthetic Ligands

Researchers worldwide attempt to identify novel compounds using in-silico methods and validate them through in vivo experiments, which were synthesized and deposited in the PubChem database.^52,53^ The protein–ligand complexes have been studied systematically using molecular dynamics simulations, metadynamics calculations, and free energy calculations,^54–56^ Using computational modeling, several small-molecule GPR17 agonists have been identified recently.^40^ They have all been experimentally validated in activating GPR17 effectively. However, none have shown notable results throughout the clinical trial stages. Thus, there is still a need to discover remarkable endogenous GPR17 ligands. Our earlier study used comparative modeling to propose a theoretical 3D structural model of the human GPR17.^41^

Among all synthetic ligands developed for GPR17, ASN02563583, and ASN04885796 exhibits the most significant documented binding affinities and selectivity.^57^ Due to their robust efficacy and elevated sensitivity in the nanomolar range, these compounds have provided researchers with dependable resources for investigating and examining the applications of GPR17. MDL29951 functions as a dual activator, mimicking both uracil nucleotides and cysteinyl-leukotrienes, though with reduced selectivity and reliability.^58^ ASN02563583 and ASN04885796 are superior prospects for preclinical testing due to their precise receptor targeting and little off-target effects. The utilization of these compounds in pharmacological research may facilitate the development of targeted treatments for neuroinflammatory and oncological disorders, particularly glioblastoma.

Our research group has synthesized the lead molecule for further evaluation and utilized this sophisticated model to predict novel ligands, such as AC1MLNKK and T0510.3657. To confirm that the synthesized compound can activate GPR17, the downstream signaling pathways of the lead chemical, including calcium and cAMP mobilization, are examined. To further understand the cytotoxicity impact of the top chemical against GBM proliferation, its capacity to block the development of GBM cells is also discussed.^59^

The most significant ligand identified to activate GPR17 (Figure 2) is the synthetic indole agonist 3-(2-carboxyethyl)-4,6-dichloro-1H-indole-2-carboxylic acid (MDL29951).^4,58^ More recently, Baqi and colleagues enhanced the efficacy of MDL29951 indole derivatives by adding various substituents to the 4- and 6-positions.^60^ The 4-position of the indole only permits the presence of smaller substituents before deteriorating or losing potency, but substituents at the 6-position of the indole may be significant and lipophilic.^40^ Considering these findings, we performed docking experiments on a library of indoline derivatives. Also, there was structural similarity of MDL29951 to a potential antitumor indoline-derived aminoalkylphenol.^40^ In addition, other groups have identified specific ligands, namely N6-cyclopentyl ATP, Galinex, and 24­(S)-hydroxycholesterol (24S-HC), that activate GPR17.^61^ The repurposed drug HAMI3379, which functions as an antagonist for the orphan G protein-coupled receptor GPR17, was identified by the Kostenis group.^62^ HAMI3379 inhibits endogenous GPR17 signaling in primary rodent oligodendrocytes, as well as in recombinant GPR17 signaling across human, rat, and mouse in various cellular settings. The therapeutic progress of GPR17 necessitates a definitive determination of whether GBM cell death is induced by agonist or antagonist mechanisms. The integration of mechanistic understanding and advancements in medicinal chemistry creates new opportunities for targeting GPR17 therapeutically in GBM. Subsequent research should elucidate the biological roles of GPR17 in glioblastoma progression and identify appropriate BBB-permeable compounds or drugs that mitigate adverse effects. While these challenges are general to GBM, the therapeutic rationale for targeting GPR17 lies in its emerging role in modulating the tumor microenvironment and glioma stem-like cell dynamics.

**Figure 2:**
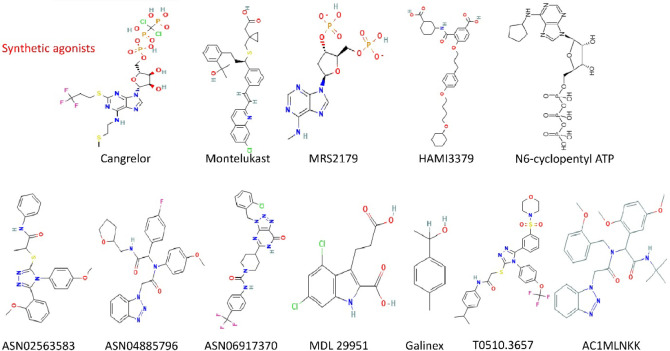
Representative structures of synthetic ligands targeting GPR17. The figure includes repurposed antagonists (e.g., cangrelor, montelukast, HAMI3379), experimental antagonists (e.g., MRS2179, N6-cyclopentyl ATP), and novel synthetic agonists (e.g., ASN02563583, ASN04885796, MDL29951, Galinex, T0510.3657, and AC1MLNKK). These compounds display structural diversity and varying degrees of selectivity and efficacy, underscoring ongoing efforts in GPR17-targeted drug discovery for oncological applications.

### 4.3 GPR17-Associated Signaling

Ongoing research is being conducted on the interactions between GPR17 proteins. Although much is still to be learned, various protein functional partners have been identified that influences GPR17’s function, trafficking, and signaling.^12^ GPR17 interacts with heterotrimeric G proteins upon ligand binding.^25^ GPR17 activation triggers the substitution of GDP with GTP on the Gα subunit of the G protein, causing the separation of the Gα and Gβγ subunits (Figure 3).^63^ These subunits further control signaling pathways, including adenylyl cyclase/cAMP route or the phospholipase C (PLC)/IP3 pathway, depending on the particular subtype of G protein involved.^64^

**Figure 3:**
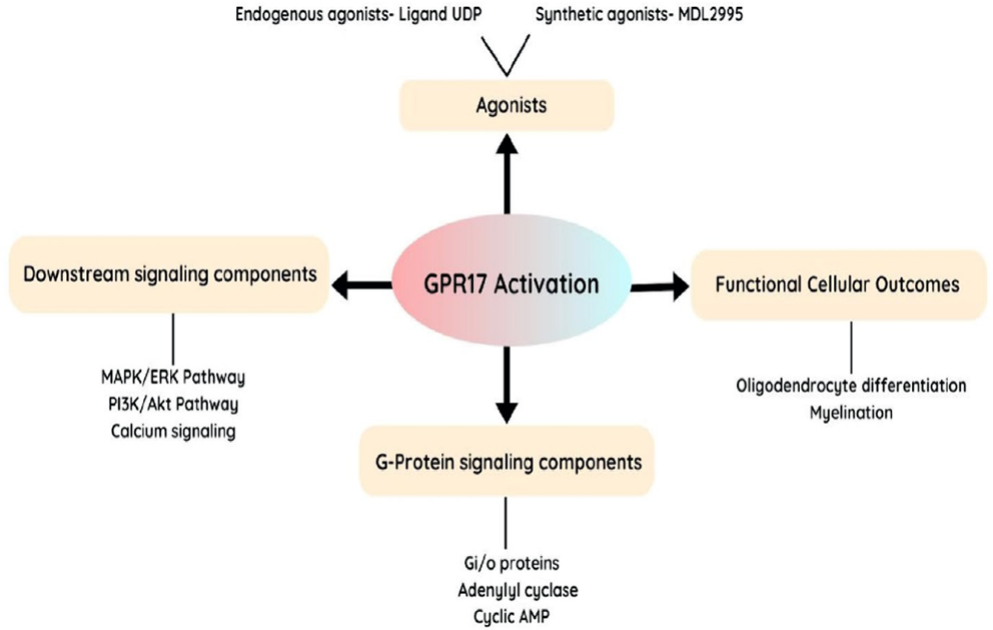
Schematic representation of GPR17 activation and its signaling pathways. GPR17 is activated by endogenous agonists (UDP) and synthetic agonists (MDL29951), leading to downstream signaling through G-protein components (Gi/o proteins, adenylyl cyclase, and cyclic AMP). Activation of GPR17 influences key signaling pathways, including the MAPK/ERK, PI3K/Akt, and calcium signaling pathways.

Recent research elucidates the internal signals of GPR17, indicating its potential as a viable therapeutic target. A regulatory link occurs between GPR17 and β-arrestins, instructing them to desensitize active GPCRs, thereby facilitating the internalization of the receptors and promoting atypical signaling. Daniele et al. (2014) demonstrated that during activation, GPR17 recruits β-arrestins, which can influence the receptor’s pathway and result in varied signaling effects.^65,66^ Furthermore, GPR17, regulated by GRK enzymes, enhances its phosphorylation and influences its interactions with the environment. GRKs also modulate the responsiveness and localization of GPR17.^67^ A supplementary regulatory mechanism is provided by the receptor’s C-terminal PDZ-binding motif which interacts with several scaffold proteins containing PDZ domains. These proteins associate GPR17 with specific biological components and alter the signaling patterns in glial cells.^68^ We currently lack a clear understanding of how to find biased synthetic compounds that preferentially activate specific signaling pathways via GPR17. While no approved biased ligands for GPR17 have been identified, preliminary pharmacological assays indicate that these receptors may exhibit biased signaling, implying that the response varies according to the ligand. Consequently, it presents the chances to identify novel pharmaceuticals for GPR17, aiding in the treatment of disorders with fewer side effects. Integrating our understanding of the GPR17 protein with current pharmacological approaches enhances our perspective on the treatment of glioblastoma.

Several regulatory proteins, like kinases, phosphatases, and scaffold proteins, can interact with GPR17 to regulate its activity and associated signaling pathways.^69^ Protein kinase A (PKA) and protein kinase C (PKC) phosphorylate GPR17, leading to receptor desensitization and internalization.^70^ GPR17 has the potential to interact with other proteins that play a role in cellular processes, such as vesicle movement, cytoskeleton dynamics, and protein folding. These interactions may impact the localization, stability, and function of GPR17.^71^ It is crucial to identify and describe the protein partners of GPR17 to comprehend the intricate signaling networks in which this receptor is involved and clarify its functions in normal and abnormal biological processes. To reveal more GPR17-protein interactions and their functional importance, additional studies must be conducted using methods such as coimmunoprecipitation, mass spectrometry, and protein–protein interaction assays.

## 5 Challenges in Treating GBM

The discovery of anti-GBM drugs faces multiple challenges, including the design of small-molecule inhibitors (SMIs) and the use of medicinal chemistry techniques to enhance treatment effectiveness, as outlined in Figure 4. The GPR17 represents a unique molecular target in glioblastoma research due to its involvement in tumor progression and migration, as well as its potential role in glioma stem-like cell populations. Pursuing innovative treatment strategies necessitates an in-depth understanding of GPR17’s activity, and the drug discovery challenges are presented.

**Figure 4:**
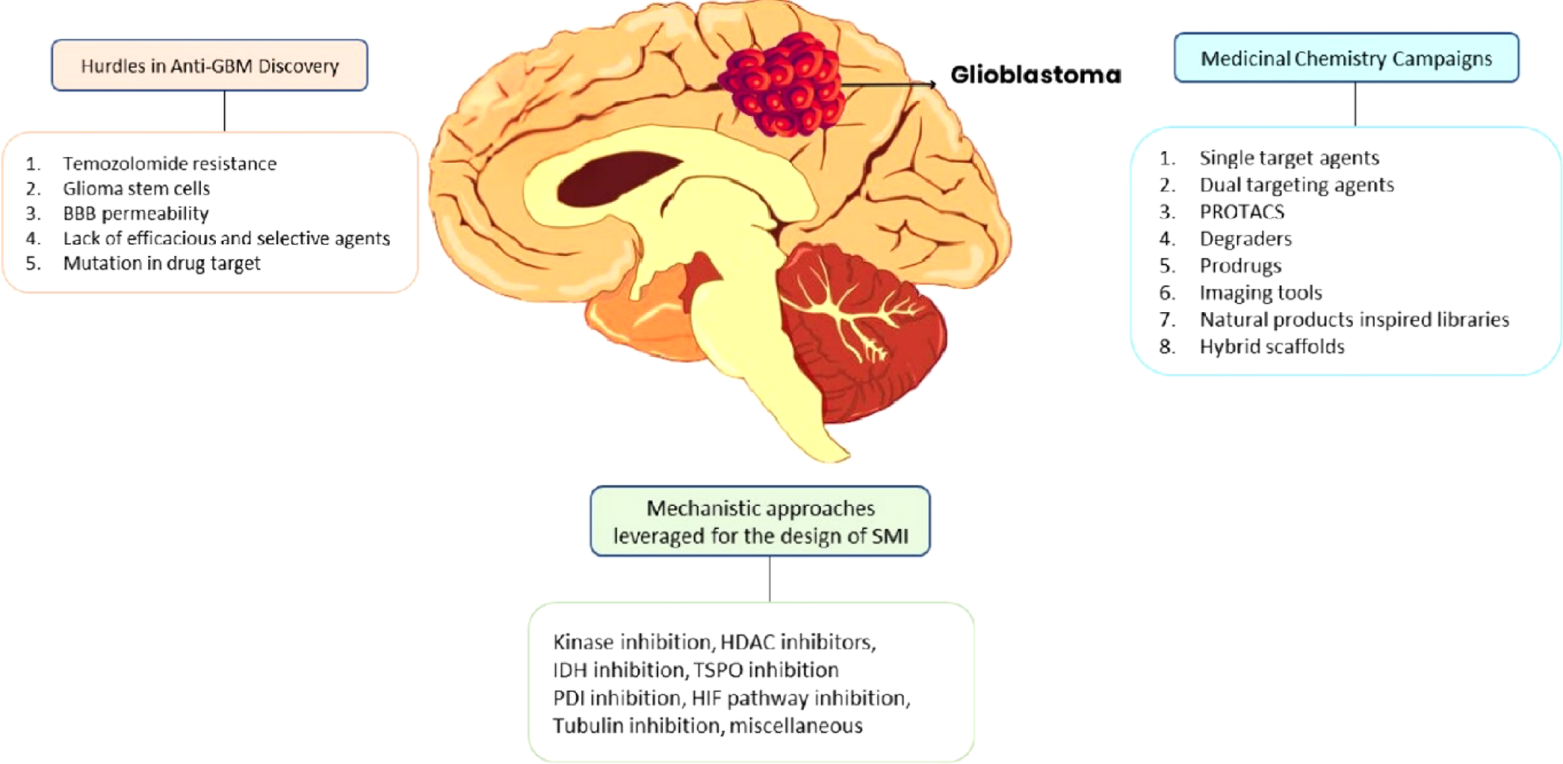
Overview of challenges and strategies in glioblastoma (GBM) drug discovery. The diagram highlights key hurdles, including Temozolomide resistance, glioma stem cells, and blood–brain barrier (BBB) permeability. Mechanistic approaches leveraged for small-molecule inhibitor (SMI) design include kinase inhibition, HDAC inhibition, IDH inhibition, and others. Various medicinal chemistry campaigns, including single- and dual-target agents, PROTACs, degraders, prodrugs, and hybrid scaffolds, are explored to enhance the efficacy of GBM treatment.

The significant obstacles researchers encounter in developing anti-GBM drugs are explicitly related to targeting GPR17. The primary obstacle in GBM therapy is the resistance to Temozolomide (TMZ), rendering it useless for many individuals due to the activation of DNA repair mechanisms and the existence of glioma stem cells.^72^ Regulating GPR17 activity may provide a novel strategy for addressing TMZ-resistant tumors, as it may safeguard glioma stem cells and lead to therapeutic ineffectiveness. The therapeutic challenges posed by glioma stem cells (GSCs) intensify because of their increased resistance to chemotherapy and radiation therapy.^73^ Future investigations into GPR17’s roles during oligodendrocyte precursor cell development warrant consideration based on established research results.

Given that GPR17 expression predominantly occurs in oligodendrocytes of the central nervous system, any therapeutic interventions targeting this receptor must address the challenge posed by the BBB. This regulation pertains to all CNS-targeting drugs broadly and specifically to GPR17-targeted drugs, as they must interact with oligodendroglial cells residing within the brain’s core.^74^ The advancement of targeted GPR17 drugs is complicated by the structural similarities between GPCRs and other proteins, which can lead to undesirable side effects. Analyzing the effects of GPR17 agonists and antagonists on GBM progression will determine the optimal therapeutic plan for patient recovery.

## 6 Conclusion

Several obstacles hinder the use of GPCR-targeted drugs for patient usage. Achieving selectivity for specific ligands is particularly challenging due to the high degree of similarity among various GPCRs in their compositions. Numerous synthetic compounds exhibit adverse effects, inadequate cerebral penetration, and lack sufficient potency. Moreover, GPR17-targeted therapies require time due to the difficulty in identifying selective pharmacological agents and the absence of clinical studies.

Small-molecule agonists or antagonists that modulate GPR17 may be feasible, and integrated approaches that combine GPR17 regulation with the suppression of BRAF (PMK/AKT) or MAPK pathways could enhance therapeutic outcomes. Future therapeutic and diagnostic advancements include innovative approaches such as Proteolysis Targeting Chimeras (PROTAC) for targeted receptor degradation and enhanced prodrug penetration of the BBB. The evaluation of GPCR-focused libraries and hybrid scaffold libraries is expected to provide promising GPR17-targeting compounds, given that many of the proposed heads are derived from natural products. While these methodologies are still theoretical, they serve as valuable recommendations for exploring innovative treatment approaches for GBM.

Advancements in structural biology, machine learning, and computational drug design have created new potential to develop GPR17 ligands with enhanced binding affinity and improved permeability across the BBB. The utilization of modern technologies and algorithms facilitates drug development through the use of 3D architectures. Contemporary models, such as patient-derived glioma organoids and xenografts are utilized to evaluate the efficacy of pharmaceuticals and the mechanisms by which tumors develop resistance. Biological substances, including monoclonal and bispecific antibodies, may assist in targeting therapy for the nervous system and in managing excessive inflammation and demyelination.

The combination of GPR17 modulators with conventional glioma treatments may lead to a more significant reduction in resistance. Incorporating spatial transcriptomics, CRISPR screening, and longitudinal imaging may provide a more accurate elucidation of the regulatory pathways of GPR17 in hypoxic and inflammatory contexts, particularly in glioma stem-like cells.

The identification of reliable biomarkers associated with GPR17 will enable healthcare providers to categorize patients more effectively and monitor the efficacy of therapy. This endeavor necessitates the rapid and effective execution of proteomic, transcriptomic, and imaging assays. A significant amount relies on transcending borders and performing research to enhance and uncover the full potential of GPR17 in these two domains.

## Data Availability

All data generated or analyzed during this study are included in this published article.

## Abbreviations

GPR17: G protein-coupled receptor 17
GPCR: G protein-coupled receptor
CysLTs: Cysteinyl leukotrienes
UDP: Uridine diphosphate
cAMP: Cyclic adenosine monophosphate
EMT: Epithelial-to-mesenchymal transition
GBM: Glioblastoma
SAGE: Serial Analysis of Gene Expression
TGF: Transforming growth factor
P2Y: Purinergic receptor family Y
TM: Transmembrane
HDAC: Histone deacetylase
IDH: Isocitrate dehydrogenase
TSPO: Translocator protein
PDI: Protein disulfide isomerase
HIF: Hypoxia-inducible factor
SMIs: Small molecule inhibitors
PROTACs: Proteolysis-targeting chimeras
PET: Positron emission tomography
MRI: Magnetic resonance imaging
MAPK: Mitogen-activated protein kinase
PLC: Phospholipase C
IP3: Inositol triphosphate
PKA: Protein kinase A
PKC: Protein kinase C
PDK1: Pyruvate dehydrogenase kinase 1
PDH: Pyruvate dehydrogenase
STAT3: Signal transducer and activator of transcription 3
Pomc: Pro-opiomelanocortin
Agrp: Agouti-related peptide
NMR: Nuclear magnetic resonance
BBB: Blood–brain barrier
ECL: Extracellular loop
ICL: Intracellular loop
TMZ: Temozolomide
OR: Olfactory receptor
Gi: Inhibitory G protein subtype
GSCs: Glioma stem cells
ERK: Extracellular signal-regulated kinase
AKT: Protein kinase B (also known as AKT)

## References

[1] Ciana P., Fumagalli M., Trincavelli M. L., Verderio C., Rosa P., Lecca D., Ferrario S., Parravicini C., Capra V., Gelosa P. (2006). The Orphan Receptor
GPR17 Identified as a New Dual Uracil Nucleotides/Cysteinyl-Leukotrienes
Receptor. EMBO J..

[2] Klauer M. J., Willette B. K. A., Tsvetanova N. G. (2024). Functional
Diversification of Cell
Signaling by GPCR Localization. J. Biol. Chem..

[3] Ou Z., Ma Y., Sun Y., Zheng G., Wang S., Xing R., Chen X., Han Y., Wang J., Lu Q. R. (2019). A GPR17-CAMP-Lactate
Signaling Axis in Oligodendrocytes Regulates
Whole-Body Metabolism. Cell Rep..

[4] Simon K., Hennen S., Merten N., Blättermann S., Gillard M., Kostenis E., Gomeza J. (2016). The Orphan
G Protein-Coupled
Receptor GPR17 Negatively Regulates Oligodendrocyte Differentiation
via Gαi/o and Its Downstream Effector Molecules*. J. Biol. Chem..

[5] Lecca D., Trincavelli M. L., Gelosa P., Sironi L., Ciana P., Fumagalli M., Villa G., Verderio C., Grumelli C., Guerrini U. (2008). The Recently Identified P2Y-like Receptor GPR17
Is a Sensor of Brain Damage and a New Target for Brain Repair. PLoS One.

[6] Yeung M. S. Y., Zdunek S., Bergmann O., Bernard S., Salehpour M., Alkass K., Perl S., Tisdale J., Possnert G., Brundin L. (2014). Dynamics of Oligodendrocyte
Generation and Myelination
in the Human Brain. Cell.

[7] Leenders F., Koole L., Slaets H., Tiane A., van den
Hove D., Vanmierlo, Vanmierlo T. (2024). Navigating Oligodendrocyte Precursor Cell Aging in
Brain Health. Mech. Ageing Dev..

[8] Angelini J., Marangon D., Raffaele S., Lecca D., Abbracchio M. (2021). The Distribution
of GPR17-Expressing Cells Correlates with White Matter Inflammation
Status in Brain Tissues of Multiple Sclerosis Patients. Int. J. Mol. Sci..

[9] Marangon D., Audano M., Pedretti S., Fumagalli M., Mitro N., Lecca D., Caruso D., Abbracchio M. P. (2022). Rewiring
of Glucose and Lipid Metabolism Induced by G Protein-Coupled Receptor
17 Silencing Enables the Transition of Oligodendrocyte Progenitors
to Myelinating Cells. Cells.

[10] Jin S., Wang X., Xiang X., Wu Y., Hu J., Li Y., Dong Y. L., Tan Y., Wu X. (2021). Inhibition of GPR17
with Cangrelor Improves Cognitive Impairment and Synaptic Deficits
Induced by Aβ1–42 through Nrf2/HO-1 and NF-ΚB Signaling
Pathway in Mice. Int. Immunopharmacol..

[11] Sood R., Anoopkumar-Dukie S., Rudrawar S., Hall S. (2024). Neuromodulatory
Effects
of Leukotriene Receptor Antagonists: A Comprehensive Review. Eur. J. Pharmacol..

[12] Fumagalli, M. ; Lecca, D. ; Coppolino, G. T. ; Parravicini, C. ; Abbracchio, M. P. Pharmacological Properties and Biological Functions of the GPR17 Receptor, a Potential Target for Neuro-Regenerative Medicine BT. In Protein Reviews; Atassi, M. Z. , Ed.; Springer Singapore: Singapore, 2017; Vol: 19, pp. 169–192. 10.1007/5584_2017_92 28828731

[13] Mutharasu G., Murugesan A., Konda Mani S., Yli-Harja O., Kandhavelu M. (2022). Transcriptomic Analysis of Glioblastoma Multiforme
Providing New Insights into GPR17 Signaling Communication. J. Biomol. Struct. Dyn..

[14] Wong T.-S., Li G., Li S., Gao W., Chen G., Gan S., Zhang M., Li H., Wu S., Du Y. (2023). G Protein-Coupled
Receptors in Neurodegenerative Diseases and Psychiatric Disorders. Signal Transduction Targeted Ther..

[15] Doan P., Nguyen P., Murugesan A., Candeias N. R., Yli-Harja O., Kandhavelu M. (2021). Alkylaminophenol
and GPR17 Agonist for Glioblastoma
Therapy: A Combinational Approach for Enhanced Cell Death Activity. Cells.

[16] Doan P., Nguyen P., Murugesan A., Subramanian K., Konda Mani S., Kalimuthu V., Abraham B. G., Stringer B. W., Balamuthu K., Yli-Harja O. (2021). Targeting Orphan G Protein-Coupled
Receptor 17 with T0 Ligand Impairs Glioblastoma Growth. Cancers.

[17] Burke L., Butler C. T., Murphy A., Moran B., Gallagher W. M., O’Sullivan J., Kennedy B. N. (2016). Evaluation of Cysteinyl Leukotriene
Signaling as a Therapeutic Target for Colorectal Cancer. Front. Cell Dev. Biol..

[18] Murugesan A., Nguyen P., Ramesh T., Yli-Harja O., Kandhavelu M., Saravanan K. M. (2022). Molecular
Modeling and Dynamics Studies
of the Synthetic Small Molecule Agonists with GPR17 and P2Y1 Receptor. J. Biomol. Struct. Dyn..

[19] Liang Y., Kang X., Zhang H., Xu H., Wu X. (2023). Knockdown
and Inhibition of Hippocampal GPR17 Attenuates Lipopolysaccharide-Induced
Cognitive Impairment in Mice. J. Neuroinflammation.

[20] Thakur A., Faujdar C., Sharma R., Sharma S., Malik B., Nepali K., Liou J. P. (2022). Glioblastoma:
Current Status, Emerging
Targets, and Recent Advances. J. Med. Chem..

[21] Steele C. D., Abbasi A., Islam S. M. A., Bowes A. L., Khandekar A., Haase K., Hames-Fathi S., Ajayi D., Verfaillie A., Dhami P. (2022). Signatures
of Copy Number Alterations in Human Cancer. Nature.

[22] Pathak A., Tomar S., Pathak S. (2023). Epigenetics and Cancer A Comprehensive
Review. Asian Pacific J. Cancer Biol..

[23] Seker-Polat F., Pinarbasi Degirmenci N., Solaroglu I., Bagci-Onder T. (2022). Tumor Cell
Infiltration into the Brain in Glioblastoma: From Mechanisms to Clinical
Perspectives. Cancers.

[24] Erices J. I., Bizama C., Niechi I., Uribe D., Rosales A., Fabres K., Navarro-Martínez G., Torres Á., San Martín R., Roa J. C. (2023). Glioblastoma
Microenvironment and Invasiveness: New Insights and Therapeutic Targets. Int. J. Mol. Sci..

[25] Calebiro D., Koszegi Z., Lanoiselée Y., Miljus T., O’Brien S. (2021). G Protein-Coupled
Receptor-G Protein Interactions: A Single-Molecule Perspective. Physiol. Rev..

[26] Cheng L., Xia F., Li Z., Shen C., Yang Z., Hou H., Sun S., Feng Y., Yong X., Tian X. (2023). Structure,
Function and Drug Discovery of GPCR Signaling. Mol Biomed..

[27] Isberg V., Mordalski S., Munk C., Rataj K., Harpsøe K., Hauser A. S., Vroling B., Bojarski A. J., Vriend G., Gloriam D. E. (2016). GPCRdb: An Information System for G Protein-Coupled
Receptors. Nucleic Acids Res..

[28] Chan W. K. B., Zhang Y. (2020). Virtual Screening of
Human Class-A GPCRs Using Ligand
Profiles Built on Multiple Ligand–Receptor Interactions. J. Mol. Biol..

[29] Dougherty J. D., Fomchenko E. I., Akuffo A. A., Schmidt E., Helmy K. Y., Bazzoli E., Brennan C. W., Holland E. C., Milosevic A. (2012). Candidate
Pathways for Promoting Differentiation or Quiescence of Oligodendrocyte
Progenitor-like Cells in Glioma. Cancer Res..

[30] Dziedzic A., Miller E., Saluk-Bijak J., Bijak M. (2020). The GPR17 Receptor—A
Promising Goal for Therapy and a Potential Marker of the Neurodegenerative
Process in Multiple Sclerosis. Int. J. Mol.
Sci..

[31] Ye F., Wong T.-S., Chen G., Zhang Z., Zhang B., Gan S., Gao W., Li J., Wu Z., Pan X. (2022). Cryo-EM Structure of
G-Protein-Coupled Receptor GPR17 in Complex
with Inhibitory G Protein. MedComm.

[32] Lu C., Dong L., Zhou H., Li Q., Huang G., Bai S. J., Liao L. (2018). G-Protein-Coupled Receptor Gpr17
Regulates Oligodendrocyte Differentiation in Response to Lysolecithin-Induced
Demyelination. Sci. Rep..

[33] Lecca D., Raffaele S., Abbracchio M. P., Fumagalli M. (2020). Regulation
and Signaling of the GPR17 Receptor in Oligodendroglial Cells. Glia.

[34] Yang D., Zhou Q., Labroska V., Qin S., Darbalaei S., Wu Y., Yuliantie E., Xie L., Tao H., Cheng J. (2021). G Protein-Coupled Receptors:
Structure- and Function-Based Drug Discovery. Signal Transduction Targeted Ther..

[35] Zhang D., Gao Z. G., Zhang K., Kiselev E., Crane S., Wang J., Paoletta S., Yi C., Ma L., Zhang W. (2015). Two Disparate Ligand-Binding Sites in the Human P2Y1
Receptor. Nature.

[36] Senthil R., Archunan G., Vithya D., Saravanan K. M. (2025). Hexadecanoic
Acid Analogs as Potential CviR-Mediated Quorum Sensing Inhibitors
in Chromobacterium Violaceum: An in Silico Study. J. Biomol. Struct. Dyn..

[37] Marucci G., Dal Ben D., Lambertucci C., Santinelli C., Spinaci A., Thomas A., Volpini R., Buccioni M. (2016). The G Protein-Coupled
Receptor GPR17: Overview and Update. ChemMedchem.

[38] Jobe A., Vijayan R. (2024). Orphan G Protein-Coupled
Receptors: The Ongoing Search
for a Home. Front. Pharmacol..

[39] Parravicini C., Ranghino G., Abbracchio M. P., Fantucci P. (2008). GPR17: Molecular Modeling
and Dynamics Studies of the 3-D Structure and Purinergic Ligand Binding
Features in Comparison with P2Y Receptors. BMC
Bioinf..

[40] Nguyen P., Doan P., Rimpilainen T., Konda Mani S., Murugesan A., Yli-Harja O., Candeias N. R., Kandhavelu M. (2021). Synthesis
and Preclinical Validation of Novel Indole Derivatives as a GPR17
Agonist for Glioblastoma Treatment. J. Med.
Chem..

[41] Saravanan K. M., Palanivel S., Yli-Harja O., Kandhavelu M. (2018). Identification
of Novel GPR17-Agonists by Structural Bioinformatics and Signaling
Activation. Int. J. Biol. Macromol..

[42] Konda
Mani S., Thiyagarajan R., Yli-Harja O., Kandhavelu M., Murugesan A. (2023). Structural Analysis of Human G-Protein-Coupled Receptor
17 Ligand Binding Sites. J. Cell. Biochem..

[43] Chen Y., Wu H., Wang S., Koito H., Li J., Ye F., Hoang J., Escobar S. S., Gow A., Arnett H. A. (2009). The
Oligodendrocyte-Specific G Protein-Coupled Receptor GPR17 Is
a Cell-Intrinsic Timer of Myelination. Nat.
Neurosci..

[44] Öz-Arslan D., Yavuz M., Kan B. (2024). Exploring Orphan GPCRs in Neurodegenerative
Diseases. Front. Pharmacol..

[45] Miralles A. J., Unger N., Kannaiyan N., Rossner M. J., Dimou L. (2023). Analysis of
the GPR17 Receptor in NG2-Glia under Physiological Conditions Unravels
a New Subset of Oligodendrocyte Progenitor Cells with Distinct Functions. Glia.

[46] Fountain S.
J., Burnstock G. (2009). An Evolutionary
History of P2X Receptors. Purinergic Signalling.

[47] Lairion F., Carbia C., Chiesa I. M., Saporito-Magriña C., Borda N., Lazarowski A., Repetto M. G. (2023). Uridine Diphosphate
Glucose (UDP-G) Activates Oxidative Stress and Respiratory Burst in
Isolated Neutrophils. Pharmaceuticals.

[48] Lecca D., Abbracchio M. P., Fumagalli M. (2021). Purinergic Receptors on Oligodendrocyte
Progenitors: Promising Targets for Myelin Repair in Multiple Sclerosis?. Front. Pharmacol..

[49] Yuan N. Y., Medders K. E., Sanchez A. B., Shah R., de Rozieres C. M., Ojeda-Juárez D., Maung R., Williams R., Gelman B. B., Baaten B. J. (2024). A Critical Role for Macrophage-Derived Cysteinyl-Leukotrienes
in HIV-1 Induced Neuronal Injury. Brain, Behav.,
Immun..

[50] Maekawa A., Balestrieri B., Austen K. F., Kanaoka Y. (2009). GPR17 Is a Negative
Regulator of the Cysteinyl Leukotriene 1 Receptor Response to Leukotriene
D4. Proc. Natl. Acad. Sci. U. S. A..

[51] Benned-Jensen T., Rosenkilde M. M. (2010). Distinct
Expression and Ligand-Binding Profiles of
Two Constitutively Active GPR17 Splice Variants. Br. J. Pharmacol..

[52] Kim S., Chen J., Cheng T., Gindulyte A., He J., He S., Li Q., Shoemaker B. A., Thiessen P. A., Yu B. (2021). PubChem
in 2021: New
Data Content and Improved Web Interfaces. Nucleic
Acids Res..

[53] Kim S., Thiessen P. A., Bolton E. E., Chen J., Fu G., Gindulyte A., Han L., He J., He S., Shoemaker B. A. (2016). PubChem Substance and Compound Databases. Nucleic
Acids Res..

[54] Zhang H., Zhang T., Saravanan K. M., Liao L., Wu H., Zhang H., Zhang H., Pan Y., Wu X., Wei Y. (2022). DeepBindBC: A Practical Deep Learning
Method for Identifying Native-like
Protein-Ligand Complexes in Virtual Screening. Methods.

[55] Zhang H., Saravanan K. M., Zhang J. Z. H. (2023). DeepBindGCN: Integrating Molecular
Vector Representation with Graph Convolutional Neural Networks for
Protein–Ligand Interaction Prediction. Molecules.

[56] Zhang H., Fan H., Wang J., Hou T., Saravanan K. M., Xia W., Kan H. W., Li J., Zhang J. Z. H., Liang X. (2024). Revolutionizing GPCR-Ligand
Predictions: DeepGPCR with Experimental
Validation for High-Precision Drug Discovery. Briefings Bioinf..

[57] Zhuo T., Zhou S., Zhang W., Lambertucci C., Volpini R. (2017). Synthesis and Ability of New Ligands
for G Protein-Coupled
Receptors 17 (GPR17). Med. Sci. Monit..

[58] Hennen S., Wang H., Peters L., Merten N., Simon K., Spinrath A., Blättermann S., Akkari R., Schrage R., Schröder R. (2013). Decoding Signaling and Function of the
Orphan G Protein–Coupled Receptor GPR17 with a Small-Molecule
Agonist. Sci. Signaling.

[59] Wang Q., Hu B., Hu X., Kim H., Squatrito M., Scarpace L., deCarvalho A. C., Lyu S., Li P., Li Y. (2017). Tumor Evolution of Glioma-Intrinsic
Gene Expression
Subtypes Associates with Immunological Changes in the Microenvironment. Cancer Cell.

[60] Baqi Y., Pillaiyar T., Abdelrahman A., Kaufmann O., Alshaibani S., Rafehi M., Ghasimi S., Akkari R., Ritter K., Simon K. (2018). 3-(2-Carboxyethyl)­Indole-2-Carboxylic
Acid Derivatives:
Structural Requirements and Properties of Potent Agonists of the Orphan
G Protein-Coupled Receptor GPR17. J. Med. Chem..

[61] Harrington A. W., Liu C., Phillips N., Nepomuceno D., Kuei C., Chang J., Chen W., Sutton S. W., O’Malley D., Pham L. (2023). Identification
and Characterization of Select Oxysterols
as Ligands for GPR17. Br. J. Pharmacol..

[62] Merten N., Fischer J., Simon K., Zhang L., Schröder R., Peters L., Letombe A.-G., Hennen S., Schrage R., Bödefeld T. (2018). Repurposing HAMI3379 to Block GPR17 and
Promote Rodent and Human Oligodendrocyte Differentiation. Cell Chem. Biol..

[63] Wu V., Yeerna H., Nohata N., Chiou J., Harismendy O., Raimondi F., Inoue A., Russell R. B., Tamayo P., Gutkind J. S. (2019). Illuminating the
Onco-GPCRome: Novel G Protein–Coupled
Receptor-Driven Oncocrine Networks and Targets for Cancer Immunotherapy. J. Biol. Chem..

[64] Campbell A. P., Smrcka A. (2018). V Targeting G Protein-Coupled Receptor
Signalling by
Blocking G Proteins. Nat. Rev. Drug Discovery.

[65] Daniele S., Trincavelli M. L., Fumagalli M., Zappelli E., Lecca D., Bonfanti E., Campiglia P., Abbracchio M. P., Martini C. (2014). Does GRK−β
Arrestin Machinery Work as
a “Switch on” for GPR17-Mediated Activation of Intracellular
Signaling Pathways?. Cell. Signalling.

[66] Kee T. R., Khan S. A., Neidhart M. B., Masters B. M., Zhao V. K., Kim Y. K., McGill
Percy K. C., Woo J.-A.-A. (2024). The Multifaceted
Functions of β-Arrestins and Their Therapeutic Potential in
Neurodegenerative Diseases. Exp. Mol. Med..

[67] Daniele S., Trincavelli M. L., Gabelloni P., Lecca D., Rosa P., Abbracchio M. P., Martini C. (2011). Agonist-Induced Desensitization/Resensitization
of Human G Protein-Coupled Receptor 17: A Functional Cross-Talk between
Purinergic and Cysteinyl-Leukotriene Ligands. J. Pharmacol. Exp. Ther..

[68] Meraviglia V., Ulivi A. F., Boccazzi M., Valenza F., Fratangeli A., Passafaro M., Lecca D., Stagni F., Giacomini A., Bartesaghi R. (2016). SNX27, a Protein Involved in down Syndrome,
Regulates GPR17 Trafficking and Oligodendrocyte Differentiation. Glia.

[69] Wang S., Wang Y., Zou S. (2022). A Glance at
the Molecules That Regulate
Oligodendrocyte Myelination. Curr. Issues Mol.
Biol..

[70] Jong, Y.-J. I. ; Harmon, S. K. ; O’Malley, K. L. ; GPCR Signaling from Intracellular Membranes. In GPCRs As Therapeutic Targets; Gilchrist, A. , Ed.; Wiley Online Library, 2022; Vol: 1, pp. 216–298. DOI: 10.1002/9781119564782.ch8.

[71] Rivera A. D., Pieropan F., Chacon-De-La-Rocha I., Lecca D., Abbracchio M. P., Azim K., Butt A. M. (2021). Functional
Genomic Analyses Highlight
a Shift in Gpr17-Regulated Cellular Processes in Oligodendrocyte Progenitor
Cells and Underlying Myelin Dysregulation in the Aged Mouse Cerebrum. Aging Cell.

[72] Singh N., Miner A., Hennis L., Mittal S. (2021). Mechanisms of Temozolomide
Resistance in Glioblastoma - a Comprehensive Review. Cancer Drug Resist..

[73] Liebelt B. D., Shingu T., Zhou X., Ren J., Shin S. A., Hu J., Ulasov I. V. (2016). Glioma Stem Cells: Signaling, Microenvironment, and
Therapy. Stem Cells Int..

[74] Boccazzi M., Macchiarulo G., Lebon S., Janowska J., Le Charpentier T., Faivre V., Hua J., Marangon D., Lecca D., Fumagalli M. (2023). G Protein-Coupled Receptor 17 Is Regulated
by WNT Pathway during Oligodendrocyte Precursor Cell Differentiation. Neurobiol. Dis..

